# Challenges for Routine Health System Data Management in a Large Public Programme to Prevent Mother-to-Child HIV Transmission in South Africa

**DOI:** 10.1371/journal.pone.0005483

**Published:** 2009-05-12

**Authors:** Kedar S. Mate, Brandon Bennett, Wendy Mphatswe, Pierre Barker, Nigel Rollins

**Affiliations:** 1 Institute for Healthcare Improvement, Cambridge, Massachusetts, United States of America; 2 Division of Global Health Equity, Harvard Medical School, Boston, Massachusetts, United States of America; 3 Department of Paediatrics and Child Health, University of KwaZulu-Natal, Durban, South Africa; 4 Department of Paediatrics, University of North Carolina, Chapel Hill, North Carolina, United States of America; 5 Department of Child and Adolescent Health and Development, World Health Organization, Geneva, Switzerland; Harvard Medical School, United States of America

## Abstract

**Background:**

Recent changes to South Africa's prevention of mother-to-child transmission of HIV (PMTCT) guidelines have raised hope that the national goal of reducing perinatal HIV transmission rates to less than 5% can be attained. While programmatic efforts to reach this target are underway, obtaining complete and accurate data from clinical sites to track progress presents a major challenge. We assessed the completeness and accuracy of routine PMTCT data submitted to the district health information system (DHIS) in three districts of Kwazulu-Natal province, South Africa.

**Methodology/Principal Findings:**

We surveyed the completeness and accuracy of data reported for six key PMTCT data elements between January and December 2007 from all 316 clinics and hospitals in three districts. Through visits to randomly selected sites, we reconstructed reports for the same six PMTCT data elements from clinic registers and assessed accuracy of the monthly reports previously submitted to the DHIS. Data elements were reported only 50.3% of the time and were “accurate” (i.e. within 10% of reconstructed values) 12.8% of the time. The data element “Antenatal Clients Tested for HIV” was the most accurate data element (i.e. consistent with the reconstructed value) 19.8% of the time, while “HIV PCR testing of baby born to HIV positive mother” was the least accurate with only 5.3% of clinics meeting the definition of accuracy.

**Conclusions/Significance:**

Data collected and reported in the public health system across three large, high HIV-prevalence Districts was neither complete nor accurate enough to track process performance or outcomes for PMTCT care. Systematic data evaluation can determine the magnitude of the data reporting failure and guide site-specific improvements in data management. Solutions are currently being developed and tested to improve data quality.

## Introduction

Recent changes to South Africa's prevention of mother-to-child transmission of HIV (PMTCT) guidelines [1], namely the addition of azidothymidine (AZT) to single dose nevirapine (NVP) prophylaxis for pregnant mothers with HIV, have raised hope that progress can be made toward reaching a national goal of reducing perinatal HIV transmission from its current rate of about 20% [2] to less than 5% [3]. The PMTCT programme relies on a sequence of diagnostic and treatment steps. Tracking each of these steps can determine how well the system is performing; i.e. that eligible HIV infected mothers are being identified, and are receiving the correct treatment [4].

Effective monitoring and supervision of health care programmes depends on complete, accurate and timely flow of data between primary health care facilities, hospitals and a central information hub [5]. For both the clinic staff and health system managers, having access to reliable data that reflects the processes of care and clinical outcomes is the first step to ensuring effective delivery of an intervention within a health care system [6]. However, data routinely collected at health care facilities and submitted to district offices is commonly stated to be unreliable [7]. We therefore assessed the completeness and accuracy of key PMTCT data elements routinely collected and reported through the District Health Information System (DHIS) of all clinics and hospitals in three districts of KwaZulu-Natal (KZN) province in South Africa.

## Methods

### Study setting and sites

KZN is the easternmost province in South Africa with a population of over 10 million. The KZN Provincial Department of Health in partnership with the University of KZN and the Institute for Healthcare Improvement has undertaken a health systems strengthening program to improve the quality of PMTCT services in the 316 health care facilities (fixed clinics, mobile clinics, community health centers, hospitals) within three districts in the Province. Data that reflects the various processes involved in the PMTCT programme are sent each month from all antenatal clinics and labor wards to District Information Offices and then to the provincial DHIS database where it is consolidated for national reporting on ART scale-up and progress on PMTCT.

### Ethics

This study was conducted according to the principles expressed in the Declaration of Helsinki. Approval for the project was given by the Biomedical Research Ethics Committee of the University of KwaZulu-Natal (BF061/08) and also by the KwaZulu Natal Department of Health Research Committee and the respective District Managers. The Biomedical Research Ethics Committee specifically considered the need for individual consent. They decided that it would be difficult to identify who among all the health workers would need to give consent for data that was non-individualised other than to specific clinic, was not patient-specific and was routine in nature i.e. this is data that is submitted by all clinics each month and eventually becomes available in the public domain. The committee decided that individual consent from health workers in individual clinics/hospitals was not required but that consent/approval for the work should be given by the district managers of each district as well as the research committee of the Provincial Department of Health.

### Assessment of Data Completeness

To assess data completeness we surveyed six PMTCT data elements (ANC Client tested for HIV, ANC Client found to be HIV Positive, CD4 Testing of HIV Positive Pregnant Women, Nevirapine dose to woman at antenatal or labour, Nevirapine dose to baby born to HIV positive woman, HIV PCR test of baby born to HIV+ women at 6 weeks or later) that were routinely reported to the DHIS, between January and December 2007, from all 316 clinics and hospitals in the three districts. Data reporting for a specific element was considered complete if a value for the element was reported to DHIS for each of the 12 months of the study period. These data were compared by district, by type of facility and over the twelve month period.

### Assessment of Data Accuracy

A subset of 86 of 300 clinics and 13 of 16 hospitals in the three Districts were identified using a computer-generated random sequence list (SPSS) for site visits to assess whether data had been collected accurately. Clinics were visited by one of two independent teams, each with two data auditors. Prior to initiating the assessment, members of the team were specifically trained and had to successfully complete mock evaluations to ensure that they fully understood the nature of the clinic registers and meaning of data elements. The quality of these independent audits was assured through rigorous daily monitoring of the survey team by a supervisor, double data capture at sites by the two team members, and random duplicate audits. At each clinic visit, copies of the monthly reports that clinics had sent to the DHIS were collected and all of the sites' PMTCT registers were reviewed. The teams collected respective clinic PMTCT data from the three month reporting period (September through November 2007), to assess monthly variation in data collection performance. The evaluation was conducted in March 2008 (at least 4 months after the end of the reporting period) to ensure that data was not “missing” due to delays in sending data from the clinics to the DHIS.

**Figure 1:**
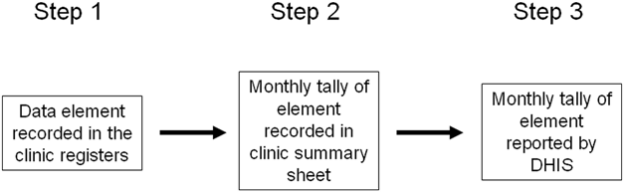
Steps for collection and reporting of PMTCT data from the clinics to the DHIS.

Data was collected from the following three sources (Figure 1): Step 1-raw data for each of the six data elements recorded in one of the numerous registers used in clinics; Step 2-the monthly tally sheet used to collate the data from the registers and then transmitted to DHIS (typically by mail or fax); Step 3-the monthly DHIS report of the 6 data elements for each clinical site recorded in the DHIS database.

Data that was “missing” altogether from either the clinics' registers or from the DHIS was excluded from the accuracy calculation. A reported data element was considered “accurate” if the value in the DHIS database was within 10% of the value reconstructed by the study teams from the registers. “Errors” were noted when reported values deviated more than 1000% from reconstructed values and also excluded from the accuracy analyses. These errors were either due to an error of data entry by the clinic staff or an error of transcription by the data auditors. Accuracy of the DHIS was also calculated as a “percentage deviation from expected” for each data element when compared to the reconstructed values obtained from the clinics' registers by the study teams.

Results were evaluated according to the role of clinics in the PMTCT programme. Many primary care clinics provide only testing and ANC care services and were expected to provide data only for HIV testing, CD4 testing, and dispensing of nevirapine, as well as HIV testing (PCR) of babies after birth. Labour ward facilities were expected to report on administration of nevirapine to mother and babies during delivery. Some facilities that were engaged in ANC care, labour and delivery as well as postnatal services were expected to report on all six indicators.

### Statistical Analysis

In order to assess the completeness of PMTCT data, Provincial DHIS monthly data reports were downloaded into a separate database, from where it was accessed for analysis. The data from the register reviews and monthly summary sheets were independently entered into the database. The data were analyzed by clinic, element type and month, using simple descriptive statistics in Microsoft Excel 2007.

## Results

### Data Completeness

**Figure 2:**
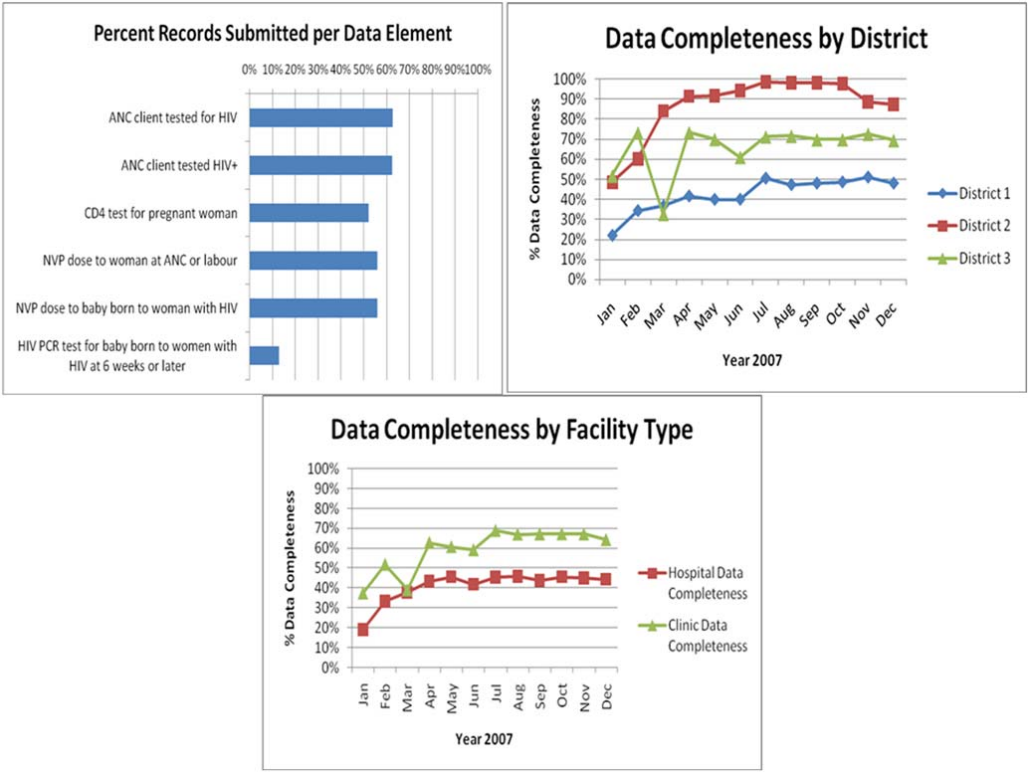
a, b & c. Completeness of individual PMTCT data elements in all districts (Figure 2a), aggregate data completeness by district for all six PMTCT data elements (Figure 2b), and aggregated data completeness by facility type for all six PMTCT data elements (Figure 2c).

There was large variation in the completeness of data reporting for each element. Analysis of all six data elements from 316 sites reported over 12 months to the DHIS showed that the data were complete only half the time (50.3%) (Figure 2a).

The best reported data element was “ANC client tested for HIV,” reported 62.7% of the time, but completely reported (i.e. every month for 12 months) by only 9.2% of clinics (n = 29). The most poorly reported data element was “PCR test to baby born of HIV positive woman at 6 weeks or later,” reported only 12.7% of the time, with no site providing complete data for every month for the 12 month study period.

These aggregate results belie significant regional, temporal and contextual variation. District 2 performed better than the others in terms of reporting on all six data elements (Figure 2b). Clinics reported more complete data when compared with larger hospitals (59.4% vs 40.9% complete, Figure 2c).

### Data Accuracy

**Figure 3:**
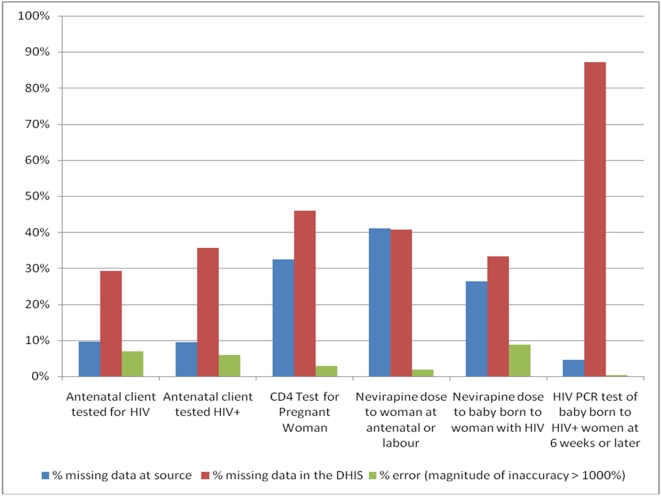
Missing data and errors according to data element.

For the six data elements studied, data was missing from the clinics' registers between 4.5% and 41.0% of the time (figure 3). Data was missing from the DHIS more often, i.e. between 29.2% and 87.1% of the time. Across all six data elements, errors (as defined above) were found to occur between 0.4% and 8.8% of the time. After the missing data and errors were excluded, the remaining data for each element (between 8% and 54% of the potential data, depending on the element) was used to determine accuracy. The initial steps of the PMTCT pathway had the most available data, with data relating to later steps in the PMTCT pathway becoming progressively less available.

Available reported data was highly inaccurate, with data elements classifiable as “accurate” only 5.3% to 19.8% of the time (Table 1). Across all six data elements, the DHIS value deviated on average by 75.2% from the corresponding values found in the clinics' registers. Compared to the other 5 elements, HIV PCR testing of infants was found to be the most accurate with an average deviation from expected of only 34.7%. However, this data element had the least available data for review. In contrast, “Antenatal Clients Tested for HIV” was the most accurate data element with 19.8% of clinics reporting data within an acceptable range.

**Table 1:** Data Accuracy by Data element shown.

	Antenatal client tested for HIV	Antenatal client tested HIV+	CD4 Test for Pregnant Woman	Nevirapine dose to woman at antenatal or labour	Nevirapine dose to baby born to woman with HIV	HIV PCR test of baby born to HIV+ women at 6 weeks or later
Average Magnitude of Deviation from expected (%)	107.7%	109.9%	80.2%	66.8%	51.7%	34.7%
% accurate (within +/−10% of expected)	19.8%	15.0%	8.1%	11.6%	17.5%	5.3%

### Data concordance between registers, clinic reports and DHIS

**Figure 4:**
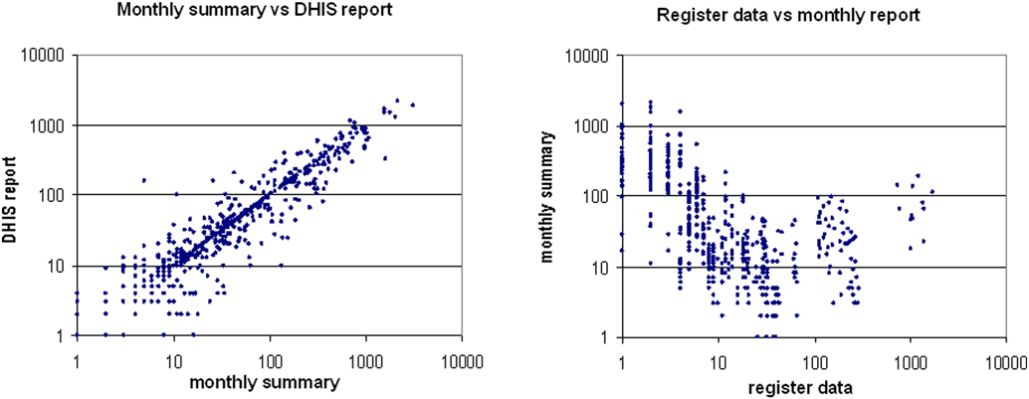
a & b: Data concordance between Clinics' Monthly Summary sheets and DHIS report (Figure 4a) and between Clinics' register data and Monthly Summary sheet (Figure 4b).

The points of ‘breakdown’ in the data transfer chain for each of the elements were determined by reconstructing data from clinic registers, and then comparing these values with the monthly reports sent to DHIS, and the values published by DHIS. Overall there was close concordance between the monthly summary sheets prepared by the clinics and the DHIS values (mean variance = 8.1, Figure 4a), whereas the clinic register values (source) and the monthly summary sheets were widely divergent (mean variance = −60.7, Figure 4b). The difference in the concordance of these 2 data sets was highly significant (p<0.00001). This observation suggests that the primary site of breakdown for accurate transfer of data is during the tallying and collation of data in the clinics before the data is sent to the DHIS.

## Discussion

Our survey shows that there are major defects in both the completeness and accuracy of the collection and reporting of data that tracks PMTCT service delivery. Yet national health systems rely on this type of data for national reports and to plan resources including financial allocations for the future. Such data might also be used to track system processes and outcomes to improve performance. This large-scale systematic review highlights the problems around the PMTCT data that are routinely collected and reported to the DHIS in South Africa, and demonstrates the need for interventions for its systematic improvement. Until these issues are addressed, routine data cannot be used to reliably inform efforts to improve PMTCT care.

The strength of this study lies in the very large sample size (all 316 clinical sites were surveyed for data completeness, 99 sites were sampled for data accuracy), the sampling technique (randomization) and the use of an objective, quality-assured “gold standard” report generated by on-site audit of the original data in clinic registers to evaluate the accuracy of PMTCT elements reported in the DHIS.

The analysis indicates that data collation at clinics, before submission to the DHIS, is the point of major breakdown that compromises data integrity. There was reasonable concordance between what the clinics actually submitted to the District Information offices for capture i.e. the Monthly summary sheets, and what subsequently appeared in the DHIS. For the six PMTCT indicators that were evaluated, the initial transfer of data from the individual clinic registers to the Monthly Summary Sheets was highly inaccurate. This may have been because numbers were incorrectly tallied, not all registers were included in the process, or register data were summated before the end of the month and therefore not all data included. By contrast, the survey team ensured that all registers were available and included, and each full month was included in their reconstruction.

The second major finding was that data were frequently not submitted by clinics to the District Information Offices for capture into the DHIS. In any health informatics system (or research study), the absence of data fundamentally weakens the accuracy and reliability of that system. Missing data, especially if substantial in quantity e.g. more than 10%, can very significantly skew results to either over-report or under-report practices or outcomes of a system. These results were presented to the district information officers responsible for the data submissions to the DHIS and to many of the clinic managers responsible for the initial data collection. Both groups initially doubted the findings presented to them. However, after investigating the indicator reports themselves, they were able to verify the findings.

Other investigators in South Africa and elsewhere have found similar defects in data systems used to track disease or public health programs. In a review of data received by the DHIS from 12 rural clinics in KZN province, the data collection process was found to be inefficient (duplication of data collection), was perceived to be a major burden on health workers, and was not being used in the clinics to improve patient care [7]. This study found a significantly lower rate of incompleteness in the DHIS records (2.5% of data missing) compared to our survey. This may reflect significant differences in the sample size (12 clinics from a single sub-district vs 316 clinics and hospitals from 3 Districts) or may reflect a specific problem related to data collection in the PMTCT programme. An evaluation of the accuracy of death notification forms in the Cape Town metropole supports the concern that defects in the public health reporting system is pervasive [8]. Errors were found in nearly all death notification forms (91%), with a major error detected in 43% of instances, resulting in documentation of an illogical sequence or cause of death.

Other reports indicate that data collected at clinic level and reported by national health programmes in developing countries cannot be relied on for disease or programme surveillance. A survey of a purpose-built data system that tracks the public sector antiretroviral treatment program in Malawi found that most clinics had complete case registration and clinical outcomes data but that case registration data were accurate in only 40% of sites [9]. The authors identified several clinics characteristics (longer experience with the ARV programme, visits by ARV supervisors, high volume of patients, dedicated clerks for recordkeeping) that were positively associated with higher data quality performance. A study of the VCT program in Kenya found major discrepancies between onsite records and those in the national office, concluding that there was significant underreporting of the data [10]. By contrast, a study that evaluated the quality of vaccination monitoring programs in 27 countries concluded that immunization rates were routinely over-reported [11]. A similar study in Mozambique found consistent over-reporting at the facility level with 44% over-reporting of BCG vaccinations and 95% over-reporting of DPT+HepB vaccinations [12]. Our study demonstrated a large variation and deficiency in the completeness and accuracy of individual data elements. This finding suggests that staff at clinics may not assign significant value to the quality of data collection and that the current data system is not used to improve the quality of PMTCT care processes at a local clinic level. Similarly, district information offices did not routinely review data completeness in order to improve the data system.

Data from multiple reports indicate that information systems in developing countries do not generally provide sufficiently useful information for effective public health management [13]. A five-country evaluation of data structures supporting health care systems in developing countries across 4 continents identified a number of structural impediments (timeliness, accuracy, simplicity, flexibility, acceptiblity, usefulness) to an effective health information system [13]. The authors proposed that while multiple problems exist, the common deficiencies were concerning the design of the system, ongoing training of personnel and dissemination of data from the system [14]. These authors recommended that after a thorough evaluation, the system should be improved through training and support.

Our survey concurs with the findings of others, that the difficulty of accurate data collection is compounded by duplication and unnecessary complexity caused by a multiplicity of registers [7]. This analysis supports the recommendations of WHO [15], [16], who argue for simplified data collection tools, a minimal common set of key indicators, reduced numbers of registers, and allocation of dedicated, trained personnel at the local level to maintain patient records and reports.

Whether current approaches to improving data systems, including further training, simplified data collecting systems, or the use of sophisticated electronic data validation systems will be sufficient to provide more reliable data remains to be determined. While undoubtedly the solution will be a multifaceted one, at least two additional principles need to be considered in any response to improving data systems: 1) Data needs to be perceived by front line clinic staff as intrinsically valuable in the management of their patients, and in the performance of their delivery of health care. This can be partially achieved through simplification of the collection and reporting process, but data will only attain significant value if it is used by clinic staff in an ongoing process to manage patients and populations. 2) Clinic staff need to be supported and supervised in the execution of data management tasks. While more accurate data may result from more rationalized data collection and reporting processes, a crucial element is to provide ongoing supervision and support to the process. Clinic managers or supervisors need to work with their local clinic staff to promote improvement of clinical practice through analysis of performance and outcomes data. Local data needs to be owned and valued by local staff, rather than relegated to the orphan status that it currently occupies.
